# A Longitudinal Study of the Impact of Loneliness on Personal Mastery Among Older Adults in Singapore

**DOI:** 10.1093/geroni/igaa057.1017

**Published:** 2020-12-16

**Authors:** Emiko Takagi, Yasuhiko Saito, Angelique W M Chan

**Affiliations:** 1 San Francisco State University, San Francisco, California, United States; 2 Nihon University, Tokyo, Japan; 3 Duke-NUS Medical School, Singapore, Singapore

## Abstract

This study uses longitudinal data to examine the association between older adults’ sense of mastery and loneliness. We examined the data of a nationally representative sample of adults 60 years and older in Singapore (Wave1, n=4,990) from the Panel of Health and Aging among Older Singaporeans Survey. The initial participants were followed up in 2011 (Wave2, n=3,103) and in 2015 (Wave3, n=1,572). At each wave, emotional loneliness was assessed using the UCLA three-item loneliness scale and sense of mastery was measured with the five items from the Pearlin Mastery Scale. We conducted cross-lagged regression analyses where loneliness and personal mastery scores in each wave were treated as endogenous variables along with covariates including demographic characteristics, health conditions, and the overall strength of social network measured by Lubben Social Network Scale. The results showed that loneliness in wave 1 and wave 2 respectively predicted a lower level of personal sense of mastery in subsequent waves. However, the other direction, the influence of personal mastery in wave 1 and wave 2 on loneliness at subsequent waves, was not significant. Furthermore, the analysis showed that older adults’ relatively strong social network was related to a lower level of loneliness and a higher sense of mastery at Wave 3. The finding suggests that loneliness plays a critical role in influencing older adults’ personal sense of mastery and that the strength of social network is an important mediator of loneliness and personal sense of mastery amongst older adults and a potential area for intervention.

